# 4-[3-(4-Methyl­piperidin-1-yl)propan­amido]­benzene­sulfonamide monohydrate

**DOI:** 10.1107/S1600536812048477

**Published:** 2012-11-30

**Authors:** Hasan Türkmen, Şerife Pınar Yalçın, Mustafa Durgun, Mehmet Akkurt

**Affiliations:** a Department of Chemistry, Faculty of Arts and Sciences, Harran University, 63300 Şanlıurfa, Turkey; b Department of Physics, Faculty of Arts and Sciences, Harran University, 63300 Şanlıurfa, Turkey; c Central Research Lab, Harran University, Osmanbey Campus, 63300 Şanlıurfa, Turkey; d Department of Physics, Faculty of Sciences, Erciyes University, 38039 Kayseri, Turkey

## Abstract

In the title compound, C_15_H_23_N_3_O_3_S·H_2_O, the piperidine ring has a chair conformation. In the crystal, the sulfonamide mol­ecules are linked by N—H⋯O hydrogen bonds, forming a layer parallel to (10-1). The layers are inter­connected *via* N—H⋯O_w_, O_w_—H⋯N and O_w_—H⋯O (w = water) hydrogen bonds, forming a three-dimensional network.

## Related literature

For inhibitors of carbonic anhydrase enzyme, inhibitors of cysteine protease enzyme, the anti­bacterial and anti­microbial activity and physical properties of sulfonamides and their derivatives and for their pharmacological applications, see: Supuran (2008 ▶); Turkmen *et al.* (2005 ▶); Rami *et al.* (2011 ▶). For related structures, see: Yalçın *et al.* (2012 ▶); Akkurt *et al.* (2010*a* ▶,*b* ▶). For puckering analysis, see: Cremer & Pople (1975 ▶).
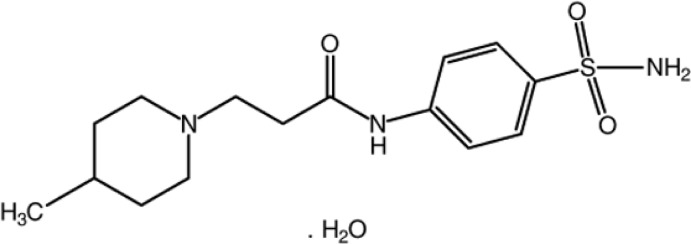


## Experimental

### 

#### Crystal data

C_15_H_23_N_3_O_3_S·H_2_O
*M* *_r_* = 343.45
Monoclinic, 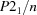

*a* = 11.3200 (3) Å
*b* = 7.4068 (3) Å
*c* = 20.7937 (8) Å
β = 96.787 (2)°
*V* = 1731.23 (11) Å^3^
*Z* = 4
Mo *K*α radiation
μ = 0.21 mm^−1^
*T* = 294 K
0.24 × 0.15 × 0.12 mm

#### Data collection

Rigaku R-AXIS RAPID-S diffractometer
Absorption correction: part of the refinement model (Δ*F*) (*XABS2*; Parkin *et al.*, 1995 ▶) *T*_min_ = 0.951, *T*_max_ = 0.975
36305 measured reflections
3540 independent reflections
2259 reflections with *I* > 2σ(*I*)
*R*_int_ = 0.166

#### Refinement

*R*[*F* ^2^ > 2σ(*F* ^2^)] = 0.073
*wR*(*F* ^2^) = 0.178
*S* = 1.02
3540 reflections
225 parameters
6 restraints
H atoms treated by a mixture of independent and constrained refinement
Δρ_max_ = 0.28 e Å^−3^
Δρ_min_ = −0.19 e Å^−3^

### 

Data collection: *CrystalClear* (Rigaku/MSC, 2005 ▶); cell refinement: *CrystalClear*; data reduction: *CrystalClear*; program(s) used to solve structure: *SIR97* (Altomare *et al.*, 1999 ▶); program(s) used to refine structure: *SHELXL97* (Sheldrick, 2008 ▶); molecular graphics: *ORTEP-3 for Windows* (Farrugia, 2012 ▶); software used to prepare material for publication: *WinGX* (Farrugia, 2012 ▶) and *PLATON* (Spek, 2009 ▶).

## Supplementary Material

Crystal structure: contains datablock(s) global, I. DOI: 10.1107/S1600536812048477/is5222sup1.cif

Structure factors: contains datablock(s) I. DOI: 10.1107/S1600536812048477/is5222Isup2.hkl

Supplementary material file. DOI: 10.1107/S1600536812048477/is5222Isup3.cml

Additional supplementary materials:  crystallographic information; 3D view; checkCIF report

## Figures and Tables

**Table 1:** Hydrogen-bond geometry (Å, °)

*D*—H⋯*A*	*D*—H	H⋯*A*	*D*⋯*A*	*D*—H⋯*A*
N1—H1*NA*⋯O1^i^	0.88 (3)	2.17 (4)	2.971 (4)	150 (4)
N1—H1*NB*⋯O3^ii^	0.87 (3)	2.18 (3)	3.035 (4)	168 (3)
N2—H2*N*⋯O1*W*	0.89 (3)	1.99 (3)	2.871 (4)	176 (3)
O1*W*—H*WA*⋯O2^iii^	0.83 (3)	2.23 (2)	3.037 (3)	165 (4)
O1*W*—H*WB*⋯N3^iv^	0.84 (2)	1.95 (2)	2.783 (4)	174 (5)

Symmetry codes: (i) 
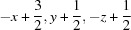
; (ii) 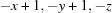; (iii) 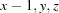; (iv) 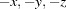.

**Fig. 1.**
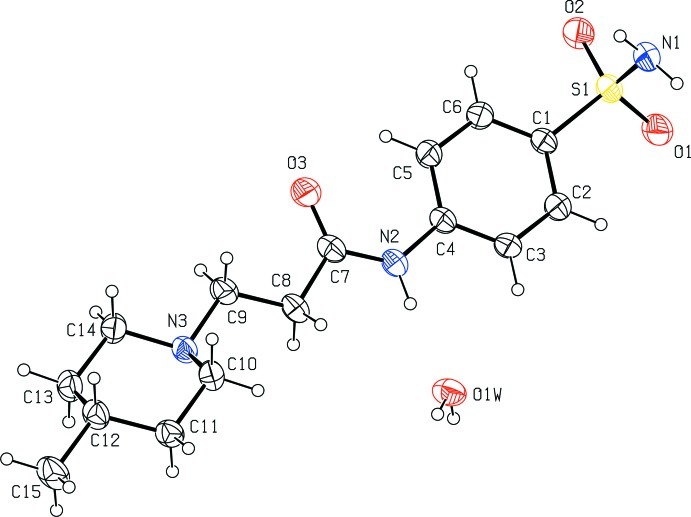
The molecular structure of the title molecule with the atom numbering scheme. Displacement ellipsoids for non-H atoms are drawn at the 30% probability level.

**Fig. 2.**
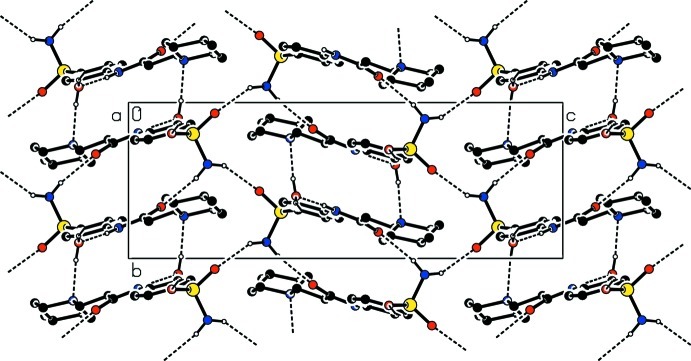
View of the packing and hydrogen bonds of the title compound down the a axis. H atoms not involved in the hydrogen bonds have been omitted for clarity.

## supplementary crystallographic information

### Comment

Sulfonamides have been used as therapeutic agents for over fifty years. The basic sulfonamide group –SO_2_NH– occurs in various biological active compounds including antimicrobial drugs, antithyroid agents, antitumor, antibiotics and inhibitors of carbonic anhydrase (Rami *et al.*, 2011; Supuran, 2008; Turkmen *et al.*, 2005). In the present study, we have prepared and determined the crystal structure of the 4-(3-methylpiperidinopropionylamino)-benzenesulfonamide.

In the title compound (Fig. 1), the piperidine ring has a chair conformation, with the puckering parameters (Cremer & Pople, 1975) of Q_T_ = 0.571 (4) Å, θ = 180.0 (4)° and φ = 150 (19)°. The O3—C7—N2—C4, O3—C7—C8—C9, N2—C7—C8—C9 and C7—C8—C9—N3 torsion angles are -0.0 (6), 2.5 (5), -178.2 (3) and 174.1 (3)°, respectively. The bond lengths and bond angles are within the normal range and are comparable to those previously reported for the related similar structures (Pınar Yalçın *et al.*, 2012; Akkurt *et al.*, 2010*a*,*b*). The crystal structure is stabilized by intermolecular N—H···O, N—H···O_water_, O_water_—H···N and O_water_—H···O hydrogen bonds (Table 1 and Fig. 2), forming a three dimensional network.

### Experimental

The starting material, 4-(3-chloropropionylamino)benzenesulfonamide, was prepared by the reaction of sulfanilamide with 3-chloropropanoylchloride (Turkmen *et al.*, 2005). The title compound, 4-(3-methylpiperidinopropionylamino)benzenesulfonamide was prepared by the reaction of 4-(3-chloropropionylamino)benzenesulfonamide with 3-methylpiperidine (Turkmen *et al.*, 2005). To a stirred solution of an excess of 3-methylpiperidine (1.12 g, 11.4 mmol) and TEA (1.75 g, 7.6 mmol) in tetrahydrofuran (20 ml) was added 4-(3-chloropropionylamino)benzenesulfonamide (1.00 g, 3.80 mmol) over 30 min at 273 K. After completion of the addition the reaction was allowed to warm to room temperature and stirred at 313 K for 48 h. The impurity was removed by flash column chromotography (ethyl acetate/methanol: 6/1) to give the title compound. (70%), m.p. 435–437 K; Anal. Calculated for C_15_H_23_N_2_O_3_S (%): C 55.36, H 7.12, N 12.91, S 9.85. Found (%): C 55.17, H 7.44, N 12.7, S 9.43; (KBr, υ_max_/cm^-1^) 1682 (NHCO), 1181 (SO_2_NH_2_); dH(DMSO-d_6_) 11.83 (1*H*, s, –CONH), 7.85 (4*H*, m, –Ar—H), 7.2 (2*H*, s, SO_2_NH_2_), 2.72 (2*H*, t, *J* 6 Hz, –NCH_2_), 2.55 (2*H*, t, *J* 6 Hz, CH_2_CO), 2.24 (4*H*, t, *J* 6 Hz, CH_2_NCH_2_), 1.67 (H, s, CH_3_CH), 1.48 (4*H*, t, *J* 6 Hz, CH_2_CHCH_2_), 1.01 (3*H*, s, CH_3_–); dC(DMSO-d_6_) 173.28 (C=O), 144.83 (CNH–),138.24 (C—SO_2_NH_2_), 128.7 (C-2 Aryl), 128.7 (C-2 Aryl), 121.18 (C-3 Aryl), 121.18 (C-3 Aryl), 55.7(CH_2_N–), 54.97 (CH_2_NCH_2_), 38.8 (CH_2_CO), 34.72 (CH_2_CHCH_2_), 32.04 (CH_2_CHCH_2_), 23.01 (CH_3_N–); m/*z* EI+ 327 [*M*]^+^. Crystals suitable for X-ray diffraction studies were grown by slow evaporation of an ethanol, chloroform, dichloromethane (4/3/3 *v*/*v*) solution of 4-(3-methylpiperidinopropionylamino)benzenesulfonamide.

### Refinement

The H atoms of the NH and NH_2_ groups and the water molecule were located from a difference Fourier map and refined with distance restraints of N—H = 0.88 (2) Å and O—H = 0.83 (2), H···H = 1.35 Å, and with *U*_iso_(H) = 1.5*U*_eq_(N,*O*). The rest H atoms were positioned geometrically, with C—H = 0.93–0.97 Å, and refined as riding with *U*_iso_(H) = 1.2 or 1.5*U*_eq_(C). Seven poorly fitted reflections (4 0 10), (-4 1 20), (-1 0 2), (3 0 14), (-5 5 15), (-6 1 23) and (0 5 6) were omitted from the refinement.

### Crystal data

**Table:**

C_15_H_23_N_3_O_3_S·H_2_O	*F*(000) = 736
*M**_r_* = 343.45	*D*_x_ = 1.318 Mg m^−^^3^
Monoclinic, *P*2_1_/*n*	Mo *K*α radiation, λ = 0.71073 Å
Hall symbol: -P 2yn	Cell parameters from 5649 reflections
*a* = 11.3200 (3) Å	θ = 2.2–26.4°
*b* = 7.4068 (3) Å	µ = 0.21 mm^−^^1^
*c* = 20.7937 (8) Å	*T* = 294 K
β = 96.787 (2)°	Needle, pale yellow
*V* = 1731.23 (11) Å^3^	0.24 × 0.15 × 0.12 mm
*Z* = 4	

### Data collection

**Table:**

Rigaku R-AXIS RAPID-S diffractometer	3540 independent reflections
Radiation source: Sealed Tube	2259 reflections with *I* > 2σ(*I*)
Graphite Monochromator monochromator	*R*_int_ = 0.166
Detector resolution: 10.0000 pixels mm^-1^	θ_max_ = 26.4°, θ_min_ = 2.9°
ω scans	*h* = −14→14
Absorption correction: part of the refinement model (Δ*F*) (*XABS2*; Parkin *et al.*, 1995)	*k* = −9→8
*T*_min_ = 0.951, *T*_max_ = 0.975	*l* = −26→26
36305 measured reflections	

### Refinement

**Table:**

Refinement on *F*^2^	Primary atom site location: structure-invariant direct methods
Least-squares matrix: full	Secondary atom site location: difference Fourier map
*R*[*F*^2^ > 2σ(*F*^2^)] = 0.073	Hydrogen site location: inferred from neighbouring sites
*wR*(*F*^2^) = 0.178	H atoms treated by a mixture of independent and constrained refinement
*S* = 1.02	*w* = 1/[σ^2^(*F*_o_^2^) + (0.0595*P*)^2^ + 1.0901*P*] where *P* = (*F*_o_^2^ + 2*F*_c_^2^)/3
3540 reflections	(Δ/σ)_max_ < 0.001
225 parameters	Δρ_max_ = 0.28 e Å^−^^3^
6 restraints	Δρ_min_ = −0.19 e Å^−^^3^

### Special details

**Table:**

Geometry. Bond distances, angles *etc*. have been calculated using the rounded fractional coordinates. All su's are estimated from the variances of the (full) variance-covariance matrix. The cell e.s.d.'s are taken into account in the estimation of distances, angles and torsion angles
Refinement. Refinement on *F*^2^ for ALL reflections except those flagged by the user for potential systematic errors. Weighted *R*-factors *wR* and all goodnesses of fit *S* are based on *F*^2^, conventional *R*-factors *R* are based on *F*, with *F* set to zero for negative *F*^2^. The observed criterion of *F*^2^ > σ(*F*^2^) is used only for calculating -*R*-factor-obs *etc*. and is not relevant to the choice of reflections for refinement. *R*-factors based on *F*^2^ are statistically about twice as large as those based on *F*, and *R*-factors based on ALL data will be even larger.

### Fractional atomic coordinates and isotropic or equivalent isotropic displacement parameters (Å^2^)

**Table:**

	*x*	*y*	*z*	*U*_iso_*/*U*_eq_	
S1	0.76956 (7)	0.20104 (14)	0.14854 (4)	0.0554 (3)	
O1	0.7834 (2)	0.0689 (4)	0.19866 (13)	0.0771 (11)	
O2	0.8487 (2)	0.1984 (4)	0.09984 (13)	0.0704 (10)	
O3	0.2781 (2)	0.3305 (4)	−0.07524 (13)	0.0760 (10)	
N1	0.7847 (3)	0.3954 (5)	0.18229 (15)	0.0626 (11)	
N2	0.2710 (2)	0.1957 (4)	0.02247 (15)	0.0547 (10)	
N3	−0.0921 (2)	0.2718 (4)	−0.12766 (13)	0.0488 (9)	
C1	0.6232 (3)	0.1876 (5)	0.10916 (16)	0.0485 (11)	
C2	0.5308 (3)	0.1398 (5)	0.14342 (18)	0.0567 (14)	
C3	0.4159 (3)	0.1440 (5)	0.11385 (17)	0.0544 (11)	
C4	0.3907 (3)	0.1938 (5)	0.04939 (17)	0.0486 (11)	
C5	0.4846 (3)	0.2404 (6)	0.01464 (18)	0.0614 (14)	
C6	0.5994 (3)	0.2372 (6)	0.04540 (17)	0.0603 (14)	
C7	0.2211 (3)	0.2596 (5)	−0.03523 (19)	0.0550 (11)	
C8	0.0882 (3)	0.2390 (5)	−0.04644 (18)	0.0598 (14)	
C9	0.0361 (3)	0.3066 (5)	−0.11224 (18)	0.0559 (11)	
C10	−0.1611 (3)	0.3832 (5)	−0.08670 (16)	0.0539 (12)	
C11	−0.2937 (3)	0.3567 (5)	−0.10375 (17)	0.0538 (11)	
C12	−0.3345 (3)	0.3988 (5)	−0.17425 (17)	0.0555 (11)	
C13	−0.2598 (3)	0.2866 (5)	−0.21563 (17)	0.0600 (14)	
C14	−0.1277 (3)	0.3160 (5)	−0.19643 (16)	0.0569 (14)	
C15	−0.4671 (3)	0.3679 (6)	−0.1923 (2)	0.0781 (16)	
O1W	0.1110 (2)	0.1006 (4)	0.11435 (15)	0.0665 (10)	
H1NA	0.756 (4)	0.405 (6)	0.2199 (13)	0.0940*	
H2	0.54640	0.10490	0.18650	0.0680*	
H1NB	0.767 (4)	0.486 (4)	0.1561 (18)	0.0940*	
H2N	0.221 (3)	0.161 (5)	0.0497 (16)	0.0820*	
H3	0.35390	0.11290	0.13740	0.0650*	
H5	0.46980	0.27310	−0.02870	0.0740*	
H6	0.66190	0.26940	0.02250	0.0720*	
H8A	0.05300	0.30530	−0.01330	0.0720*	
H8B	0.06800	0.11260	−0.04240	0.0720*	
H9A	0.04970	0.43560	−0.11440	0.0670*	
H9B	0.07780	0.24960	−0.14500	0.0670*	
H10A	−0.14170	0.50940	−0.09210	0.0650*	
H10B	−0.13900	0.35160	−0.04160	0.0650*	
H11A	−0.33580	0.43430	−0.07650	0.0640*	
H11B	−0.31390	0.23260	−0.09480	0.0640*	
H12	−0.31800	0.52650	−0.18170	0.0670*	
H13A	−0.27830	0.15980	−0.21090	0.0720*	
H13B	−0.27990	0.31890	−0.26080	0.0720*	
H14A	−0.08310	0.24110	−0.22330	0.0680*	
H14B	−0.10820	0.44110	−0.20410	0.0680*	
H15A	−0.48630	0.24520	−0.18290	0.1180*	
H15B	−0.48760	0.39080	−0.23770	0.1180*	
H15C	−0.51100	0.44810	−0.16770	0.1180*	
HWA	0.044 (2)	0.144 (5)	0.115 (2)	0.1000*	
HWB	0.109 (4)	−0.011 (3)	0.121 (2)	0.1000*	

### Atomic displacement parameters (Å^2^)

**Table:**

	*U*^11^	*U*^22^	*U*^33^	*U*^12^	*U*^13^	*U*^23^
S1	0.0363 (5)	0.0734 (7)	0.0547 (5)	0.0018 (4)	−0.0026 (4)	0.0068 (5)
O1	0.0563 (16)	0.089 (2)	0.0806 (19)	−0.0044 (15)	−0.0147 (14)	0.0295 (16)
O2	0.0380 (13)	0.107 (2)	0.0673 (17)	0.0088 (14)	0.0107 (12)	−0.0009 (15)
O3	0.0451 (15)	0.109 (2)	0.0711 (18)	−0.0076 (14)	−0.0051 (13)	0.0250 (17)
N1	0.0512 (18)	0.081 (2)	0.054 (2)	−0.0091 (17)	−0.0008 (15)	−0.0017 (18)
N2	0.0377 (15)	0.069 (2)	0.0554 (18)	−0.0023 (15)	−0.0034 (13)	0.0039 (16)
N3	0.0367 (14)	0.0587 (18)	0.0497 (16)	−0.0001 (13)	0.0000 (12)	−0.0014 (14)
C1	0.0325 (16)	0.059 (2)	0.053 (2)	0.0016 (15)	0.0013 (14)	0.0013 (17)
C2	0.045 (2)	0.072 (3)	0.052 (2)	−0.0018 (17)	0.0017 (16)	0.0059 (18)
C3	0.0368 (18)	0.071 (2)	0.055 (2)	−0.0057 (16)	0.0043 (15)	0.0054 (18)
C4	0.0366 (17)	0.054 (2)	0.054 (2)	−0.0007 (15)	0.0002 (15)	−0.0005 (16)
C5	0.0390 (19)	0.094 (3)	0.050 (2)	−0.0023 (18)	0.0004 (16)	0.0091 (19)
C6	0.0392 (19)	0.092 (3)	0.050 (2)	−0.0019 (18)	0.0061 (16)	0.007 (2)
C7	0.0412 (19)	0.058 (2)	0.064 (2)	−0.0029 (16)	−0.0013 (17)	−0.0011 (19)
C8	0.0396 (19)	0.073 (3)	0.064 (2)	−0.0046 (17)	−0.0061 (17)	−0.0003 (19)
C9	0.0381 (18)	0.066 (2)	0.062 (2)	−0.0012 (16)	−0.0011 (16)	0.0053 (19)
C10	0.050 (2)	0.062 (2)	0.048 (2)	0.0020 (17)	−0.0018 (16)	−0.0053 (17)
C11	0.0415 (18)	0.061 (2)	0.059 (2)	0.0008 (16)	0.0059 (16)	−0.0015 (18)
C12	0.0444 (19)	0.061 (2)	0.058 (2)	0.0043 (17)	−0.0066 (16)	0.0040 (18)
C13	0.053 (2)	0.074 (3)	0.050 (2)	0.0034 (19)	−0.0063 (17)	0.0028 (18)
C14	0.052 (2)	0.074 (3)	0.0439 (19)	0.0041 (18)	0.0025 (16)	0.0021 (18)
C15	0.043 (2)	0.095 (3)	0.092 (3)	0.003 (2)	−0.010 (2)	0.007 (3)
O1W	0.0476 (14)	0.0696 (18)	0.0827 (19)	−0.0055 (13)	0.0089 (14)	0.0065 (16)

### Geometric parameters (Å, º)

**Table:**

S1—O1	1.425 (3)	C11—C12	1.516 (5)
S1—O2	1.429 (3)	C12—C15	1.521 (5)
S1—N1	1.602 (4)	C12—C13	1.523 (5)
S1—C1	1.762 (3)	C13—C14	1.517 (5)
O3—C7	1.229 (5)	C2—H2	0.9300
O1W—HWA	0.83 (3)	C3—H3	0.9300
O1W—HWB	0.84 (2)	C5—H5	0.9300
N2—C4	1.404 (4)	C6—H6	0.9300
N2—C7	1.350 (5)	C8—H8A	0.9700
N3—C9	1.472 (4)	C8—H8B	0.9700
N3—C14	1.476 (4)	C9—H9A	0.9700
N3—C10	1.474 (4)	C9—H9B	0.9700
N1—H1NA	0.88 (3)	C10—H10B	0.9700
N1—H1NB	0.87 (3)	C10—H10A	0.9700
N2—H2N	0.89 (3)	C11—H11A	0.9700
C1—C2	1.380 (5)	C11—H11B	0.9700
C1—C6	1.372 (5)	C12—H12	0.9800
C2—C3	1.372 (5)	C13—H13B	0.9700
C3—C4	1.387 (5)	C13—H13A	0.9700
C4—C5	1.397 (5)	C14—H14B	0.9700
C5—C6	1.379 (5)	C14—H14A	0.9700
C7—C8	1.503 (5)	C15—H15C	0.9600
C8—C9	1.510 (5)	C15—H15A	0.9600
C10—C11	1.514 (5)	C15—H15B	0.9600
			
O1—S1—O2	119.01 (16)	C6—C5—H5	120.00
O1—S1—N1	107.46 (17)	C5—C6—H6	119.00
O1—S1—C1	108.49 (16)	C1—C6—H6	119.00
O2—S1—N1	106.21 (18)	C7—C8—H8A	109.00
O2—S1—C1	107.68 (16)	C9—C8—H8A	109.00
N1—S1—C1	107.48 (18)	C9—C8—H8B	109.00
HWA—O1W—HWB	110 (4)	H8A—C8—H8B	108.00
C4—N2—C7	129.9 (3)	C7—C8—H8B	109.00
C9—N3—C14	108.8 (3)	N3—C9—H9B	109.00
C10—N3—C14	109.5 (3)	C8—C9—H9A	109.00
C9—N3—C10	110.6 (3)	N3—C9—H9A	109.00
S1—N1—H1NA	115 (3)	H9A—C9—H9B	108.00
H1NA—N1—H1NB	114 (4)	C8—C9—H9B	109.00
S1—N1—H1NB	114 (2)	N3—C10—H10B	109.00
C4—N2—H2N	114 (2)	C11—C10—H10A	109.00
C7—N2—H2N	116 (2)	C11—C10—H10B	109.00
C2—C1—C6	119.6 (3)	H10A—C10—H10B	108.00
S1—C1—C2	120.2 (3)	N3—C10—H10A	109.00
S1—C1—C6	120.0 (3)	C10—C11—H11A	109.00
C1—C2—C3	120.0 (3)	C10—C11—H11B	109.00
C2—C3—C4	121.0 (3)	C12—C11—H11B	109.00
N2—C4—C5	123.3 (3)	H11A—C11—H11B	108.00
N2—C4—C3	117.8 (3)	C12—C11—H11A	109.00
C3—C4—C5	118.9 (3)	C11—C12—H12	108.00
C4—C5—C6	119.3 (3)	C15—C12—H12	108.00
C1—C6—C5	121.3 (3)	C13—C12—H12	108.00
O3—C7—C8	122.3 (3)	C12—C13—H13A	109.00
O3—C7—N2	123.6 (3)	C14—C13—H13A	109.00
N2—C7—C8	114.1 (3)	C14—C13—H13B	109.00
C7—C8—C9	112.6 (3)	H13A—C13—H13B	108.00
N3—C9—C8	114.1 (3)	C12—C13—H13B	109.00
N3—C10—C11	111.8 (3)	N3—C14—H14A	109.00
C10—C11—C12	112.3 (3)	C13—C14—H14A	109.00
C13—C12—C15	112.1 (3)	C13—C14—H14B	109.00
C11—C12—C13	107.9 (3)	N3—C14—H14B	109.00
C11—C12—C15	112.7 (3)	H14A—C14—H14B	108.00
C12—C13—C14	111.7 (3)	C12—C15—H15B	109.00
N3—C14—C13	111.8 (3)	C12—C15—H15C	109.00
C1—C2—H2	120.00	C12—C15—H15A	109.00
C3—C2—H2	120.00	H15A—C15—H15C	109.00
C4—C3—H3	119.00	H15B—C15—H15C	110.00
C2—C3—H3	120.00	H15A—C15—H15B	109.00
C4—C5—H5	120.00		
			
O1—S1—C1—C2	32.8 (4)	S1—C1—C6—C5	−175.3 (3)
O2—S1—C1—C2	162.8 (3)	S1—C1—C2—C3	174.6 (3)
N1—S1—C1—C2	−83.2 (3)	C1—C2—C3—C4	0.8 (6)
O1—S1—C1—C6	−151.9 (3)	C2—C3—C4—N2	−179.7 (3)
O2—S1—C1—C6	−21.9 (4)	C2—C3—C4—C5	−0.1 (6)
N1—S1—C1—C6	92.2 (3)	N2—C4—C5—C6	179.0 (4)
C7—N2—C4—C3	170.6 (4)	C3—C4—C5—C6	−0.6 (6)
C7—N2—C4—C5	−9.1 (6)	C4—C5—C6—C1	0.6 (6)
C4—N2—C7—O3	0.0 (6)	N2—C7—C8—C9	−178.2 (3)
C4—N2—C7—C8	−179.3 (3)	O3—C7—C8—C9	2.5 (5)
C9—N3—C10—C11	177.3 (3)	C7—C8—C9—N3	174.1 (3)
C10—N3—C9—C8	69.1 (4)	N3—C10—C11—C12	−57.4 (4)
C14—N3—C9—C8	−170.7 (3)	C10—C11—C12—C15	178.5 (3)
C10—N3—C14—C13	−58.0 (4)	C10—C11—C12—C13	54.1 (4)
C14—N3—C10—C11	57.5 (4)	C11—C12—C13—C14	−54.3 (4)
C9—N3—C14—C13	−179.0 (3)	C15—C12—C13—C14	−179.0 (3)
C6—C1—C2—C3	−0.7 (6)	C12—C13—C14—N3	58.0 (4)
C2—C1—C6—C5	0.0 (6)		

### Hydrogen-bond geometry (Å, º)

**Table:**

*D*—H···*A*	*D*—H	H···*A*	*D*···*A*	*D*—H···*A*
N1—H1*NA*···O1^i^	0.88 (3)	2.17 (4)	2.971 (4)	150 (4)
N1—H1*NB*···O3^ii^	0.87 (3)	2.18 (3)	3.035 (4)	168 (3)
N2—H2*N*···O1*W*	0.89 (3)	1.99 (3)	2.871 (4)	176 (3)
O1*W*—H*WA*···O2^iii^	0.83 (3)	2.23 (2)	3.037 (3)	165 (4)
O1*W*—H*WB*···N3^iv^	0.84 (2)	1.95 (2)	2.783 (4)	174 (5)

Symmetry codes: (i) −*x*+3/2, *y*+1/2, −*z*+1/2; (ii) −*x*+1, −*y*+1, −*z*; (iii) *x*−1, *y*, *z*; (iv) −*x*, −*y*, −*z*.
